# “I Should at Least Have the Feeling That It […] Really Comes from Within”: Professional Nursing Views on Assisted Suicide

**DOI:** 10.1089/pmr.2023.0019

**Published:** 2023-07-21

**Authors:** Lena Dörmann, Friedemann Nauck, Karin Wolf-Ostermann, Henrikje Stanze

**Affiliations:** ^1^ Department of Social Sciences, University of Applied Sciences in Bremen, Bremen, Germany.; ^2^ Department of Palliative Medicine, University Medical Centre in Göttingen, Göttingen, Germany.; ^3^ Department of Nursing Science Research, University of Bremen, Bremen, Germany.; ^4^ Department of Social Sciences, University of Applied Sciences in Bremen, Bremen, Germany.

**Keywords:** assisted suicide, attitudes, euthanasia, grounded theory, nursing, perspectives

## Abstract

**Background:**

Due to a decision by the German Federal Constitutional Court of February 26, 2020, it is currently possible in Germany to avail of assisted suicide. The ruling has given rise to a controversial debate within the professional community as well as in society in general. Within this debate, little attention has been given to the role of nursing staff in assisted suicide. However, international studies show that nurses play an important role in assisted suicide.

**Objective:**

The aim of this study is to assess the views and attitudes of nurses from different care settings in Germany toward assisted suicide.

**Design:**

A qualitative research design was chosen to capture the subjective experience of nursing staff on suicide assistance. This was analyzed using the grounded theory method.

**Methods:**

With the help of a semi-structured, narrative-generating interview guide, in which five case vignettes are integrated, 20 interviews were conducted with nursing professionals from different care settings throughout Germany.

**Results:**

The analyzed phenomenon shows that nursing professionals need to understand the desire to die for themselves. The individual life situation of the respective patient is decisive. The action strategy based on being able to tolerate the wish to die determines how intensively they want to be involved before, during, and after assisted suicide. For nurses, however, it is undisputed that it is their professional role to accompany the patient in their “existence” and thus also in the context of assisted suicide.

**Conclusion:**

In view of the future development of assisted suicide in Germany, it seems necessary to prepare nursing professionals for activities related to assisted suicide by means of a curricular offer. In addition, nursing professionals should be supported in forming their own attitude to the issue.

## Introduction

In February 2020, a ruling by the Federal Constitutional Court (Bundesverfassungsgericht, BVerfG) led to a change in the legal situation regarding assisted suicide in Germany. Section 217 of the German Penal Code (Strafgesetzbuch), which previously criminalized the provision of assisted suicide services, was interpreted as unconstitutional by the BVerfG ruling.

The BVerfG emphasizes that there is a right to self-determined life and thus also the right to a self-determined death.^1^ As a result, every person in Germany currently has the possibility to avail of assisted suicide where this is offered by a third party. The BVerfG ruling and the resulting social and political changes have given rise to a critical discussion within society and among experts.^2^ On the one hand, the BVerfG ruling is understood as an opening toward a more liberal society.

On the other hand, the dangers that this opening can bring with it are being questioned critically. One example of such a danger is that vulnerable groups, such as people in need of care, are exposed to external and internal pressure not to burden relatives or society if there is a legal and social regulation on assisted suicide.^2^ Further, the discussion is characterized by ethical values and norms as well as by concepts of “‘self-determination’, ‘realisation of free will’ and ‘self-determined dying’”^2^ (original quote in German).

In this context, the question of the possibilities and limits of arranging a dignified and self-determined death, which cannot always be fulfilled, such as the place and time of death, is also discussed.^3^ Within the discussion, the possibilities of health care in Germany with regard to assisted suicide are also addressed. Doctors are primarily involved in the discussion as a professional group, since they are the only professional group in Germany allowed to prescribe medication.

However, international studies from countries where assisted suicide and, in some cases, killing on request are under certain conditions already possibly show that nursing professionals play an essential role in assisted suicide.^4,5^ Currently, there are no comparable scientific findings for Germany, although the German Nursing Council (Deutscher Pflegerat) stated in its 2015 policy paper that nursing professionals are particularly frequently confronted with desires to die or requests for assisted suicide. These situations often create serious dilemmas for care professionals.^6^

In the context of executed assisted suicide, situations may arise in which nursing expertise is required. This is necessary, for example, when assisted suicide leads to side effects such as convulsions or vomiting, as well as the delayed onset of death.^7^ These unpredictable situations can be very challenging for patients and their relatives as well as for the nurses providing care, and they necessitate a prior examination of the topic as well as one's own positioning within the professional group.

This requires knowledge about the subjective views and attitudes of nursing professionals toward assisted suicide as well as their possible action strategies and a prior, already formed awareness of their responsibility in assisted suicide situations. This knowledge makes it possible to better understand the field of nursing activity within assisted suicide in Germany and, based on this, to identify consequences for the professional field as well as for the institutions involved. Further, the results can also provide insights for nursing professionals outside Germany, for example, in the United States, Canada, the United Kingdom, and Netherlands.

Theory building should help to show what motivates nurses to provide support for an assisted suicide or not. In addition, it may be possible to map which tasks they consider relevant from a professional perspective to adequately provide support to the affected person and their relatives (before and after the assisted suicide).

Objectives of the SEILASS-Study (SEILASS—Sichtweisen und Einstellungen von Pflegefachkräften unterschiedlicher Versorgungssettings zur Suizidassistenz [*views and attitudes of nurses from different care settings toward suicide assistance*]):

- What are the views and attitudes of nurses in different care settings toward assisted suicide?
- What strategies for action do they have?

## Materials and Methods

### Design

To address the research question, a qualitative research method with an explorative study design was chosen. To identify the views and attitudes of nurses in different care settings toward assisted suicide, the subjective experience of the nurses, their perspectives, as well as their actions, strategies, and everyday professional situations are to be analyzed. For this purpose, it is necessary to gain a deeper insight into the subjective senses of the nursing professionals. To enable this, the grounded theory method according to Strauss (1991) and Strauss and Corbin (1996) was chosen.^8,9^

### Ethical aspects

The study was approved by the Ethics Committee of the German Society for Nursing Science (Ethikkommission der Deutschen Gesellschaft für Pflegewissenschaft) (Application No. 21-036). Participating nurses received written informed assurance stating that all personal data would be pseudonymized, that participation was voluntary and that they could withdraw at any time without giving a reason.

### Data collection

The views and attitudes of nurses from different care settings toward assisted suicide are understood as a social phenomenon that is shaped by subjective views. To access subjective perspectives, it is recommended to use narrative interviews.^10,11^ For this purpose, a semi-structured, narrative-generating interview guide with five case vignettes was developed.

For recruitment purposes, an information flyer was sent throughout Germany via the e-mail distribution lists of the German Society for Palliative Medicine (Deutsche Gesellschaft für Palliativmedizin) and the Bremen University of Applied Sciences (Hochschule Bremen). Nursing professionals could contact the study team if they were interested in an interview.

Initially, there were a few inclusion criteria. The only prerequisite was that the nursing professionals were of adult age and able to give consent and, due to the emotionality of the subject, that there was no current case of bereavement or suicide in their immediate surroundings. As the study progressed, the theoretical sampling inherent in the grounded theory was then oriented to the previously collected data within the framework of minimum and maximum contrasting.^8,9,12^

A total of 20 interviews with an average duration of 75 minutes were conducted by video telephony between January and March 2022. The interviews were conducted by two people (L.D., H.S.). The semi-structured, narrative-generating interview guide ensured a coherent structure during the interviews. All interviews were audiotaped and transcribed, verbatim and unpolished.

Interviews from the second interviewer (H.S.) were transcribed by the first interviewer (L.D.) to prove conformity. Memos were taken by the interviewer both during and immediately after the interviews. Data collection was terminated as soon as no new findings emerged in the analysis (theoretical saturation).^8,9^

### Data evaluation

The data were evaluated according to the grounded theory method of Strauss and Corbin.^8,9^ For this purpose, categories were derived from the data. The three coding phases of open, axial, and selective coding support the identification from the data of the core categories and the key category in an iterative process. The identified core categories were then arranged around the key category in the coding paradigm developed by Strauss^8^ and Strauss and Corbin.^9^

In this process, the core categories were assigned to the following component ends of the coding paradigm and thus related to each other: causal conditions, context, intervening conditions, strategies, and consequences.^9^ To ensure theoretical sensitivity, interdisciplinary research workshops were conducted at regular intervals, at which interview passages as well as categories were presented and discussed. The software MAXQDA22 was used in the computer-assisted data evaluation.

## Results

Within the framework of the study, a total of 20 nursing professionals (14 female, 6 male) from different care settings (outpatient and inpatient) were interviewed. The average age was 44.8 years. The average professional experience of the nurses was 21.2 years. Further details on the sample are described in Table 1. The nursing professionals interviewed had contact with the subjects of end of life, dying, and death.

**Table 1.** Characteristics of the Study Participants

	Number of interviewees female/male
Participants female/male	14/6
Age, years	
25–35	7/0
36–45	1/0
46–55	3/4
56–65	3/2
Denomination	
Christian–Catholic	5/4
Christian–Protestant	2/1
None	7/1
Professional training qualification in nursing (multiple answers possible)	
Vocational/workplace (apprenticeship)	8/3
Vocational/school (vocational school, commercial school)	3/2
College/university	2/0
Vocational or technical academy	6/1
Technical college	0/1
Occupational group	
Care of the elderly	0/0
Health care and nursing	13/6
Health care and pediatric nursing	1/0
Care assistance	0/0
Occupational years	
0–10	7/0
11–20	1/0
21–30	3/4
31–40	3/2
Medical fields of activity (multiple answers possible)	
Intensive care/intermediate care/emergency room	4/4
Operating theater/anesthesia	3/1
Psychiatry	4/0
Genecology/obstetrics	1/0
Internal medicine	6/2
Neurology	1/2
Palliative care	10/5
Oncology	4/0
Geriatrics	2/0
Surgery	1/2
Outpatient care	4/2
Home respiratory care	1/0
Tropical medicine/infectiology	0/1
Medical outpatient without limitation	1/0
Function (multiple answers possible)	
Health care worker and nurse	11/4
Care services	2/1
Clinic/area nursing manager	0/1
Practice supervisor	4/1
Case manager	0/1
Coordinator	1/1
Lecturer for training and further education	2/2
Bereavement counselor	1/0
Academic degree in nursing	
No	9/4
Nursing management	1/1
Pedagogy for vocational schools	1/0
Nursing science	2/0
Not specified	1/1
Work in a specialized area of palliative care (one multiple answer)	
No	4/1
Palliative care unit	4/3
Outpatient hospice services	2/1
Special outpatient palliative care (SAPV)	5/1

Following the coding paradigm, the phenomenon and other central categories were identified. The main phenomenon for nursing staff when it comes to professional work in assisted suicide appears to be the “ability to understand the desire to die.” The cause of this is a vacillating attitude toward the topic, which leads to nursing professionals wanting to assess the patient's situation individually.

On this basis, they want to improve knowledge about assisted suicide to be able to educate about the risks and consequences, and to be able to act professionally in each vulnerable situation of assisted suicide. This leads to the strategy of being able to tolerate the wish to die, irrespective of one's own attitude in favor of or against assisted suicide as such, which, in turn, leads to the consequence of always accompanying people in their “existence,” just as they are. For nurses, the intensity of professional support before, during, and after assisted suicide appears to depend on how the desire to die can be understood in relation to the individual life situation.

### Phenomenon: Being able to understand the desire to die

The main phenomenon was shown to be “being able to understand the desire to die.” This seems to be essential for nurses to be able to deal mentally with assisted suicide. The primary concern for nurses is to understand the desire to die and the wish for assisted suicide. This understanding is derived from the circumstances in which the patient finds themselves. The nurse apparently gains understanding by seeing the life and its quality as being so severely impaired that even from a professional perspective he or she can understand these circumstances and the wish to end life.

“Yes, exactly, I would say that, em, in such a situation I would not, of course, I would not just do it, I would have to know the patient and know them well and also know, em, the wishes, also those of the relatives and especially those of the patient and then, em, I believe that one can then decide to do it” (IP3)

“So, em, (4) as I said, professional support yes: kinda sorta ((laughs)) well, I would have to fully support the idea, exactly, so that's why I can do it, I think it's something where one always (3) really, well one can't, I can't say in general, I think I have to, em, decide from patient to patient, from situation to situation, so it's, em, I—after all, it's quite a step, somehow, and I am involved in it and I must be able to support it, so therefore I must, em, must be able to understand it” (IP10)

**FIG. 1.**
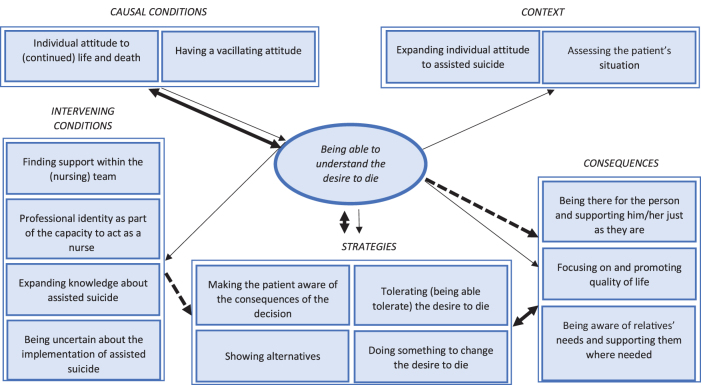
Views and attitudes of nursing professionals toward assisted suicide.

At the same time, other conditions also play a role in the phenomenon (Fig. 1).

### Causal condition: Having a vacillating attitude

One of the causes for the central phenomenon is that the nursing professionals interviewed here stated that they had a vacillating attitude toward assisted suicide. It was found that although nurses thought they had a clear stance on the issue, during the interviews they could not justify either a clear endorsement or a clear rejection of assisted suicide. Rather, they moved on a continuum that shows their own attitude to be “rather in favour” or “rather against.”

This explains why the phenomenon of “being able to understand the desire to die” is triggered by developing an attitude toward the respective situation to be able to position themselves. Based on this, nursing professionals apparently want to make themselves aware of whether providing support—whether beforehand, during, and/or afterward—is deemed possible in the framework of assisted suicide.

“That's why I think it's a question where you have to develop your own attitude and simply be clear about your own spiritual or religious orientation whether you can do it or whether you just can't do it, whether there's something standing in the way” (IP12)

In addition, it was possible to analyze that the interviewees noted during the interviews that they were currently vulnerable on their own attitude. Little or no personal experience with assisted suicide and personal life experiences ensure that the perspective on assisted suicide can change and thus that the existing attitude can be described as wavering, in the sense of being unstable.

“Well, em (4) I (2), so when I think back on it, em, or think about the topic as a whole, em, I have to say that for me my feelings on it go back and forth, em, it's not as if I was suddenly clear about it and then always remained clear, but rather that it, em, changed again and again, em, such that when I was younger, em, a young person or also as a young nurse I actually thought yes, that is understandable, em, and, em, one could do it, em, because I probably had not fully thought through and understood, em (3) the full consequences of the decision, and that changed then, so that it is now something that I, em, now rather reject or I think, em, I can't imagine how I can do such a thing in such a way that I can reconcile it well with my conscience, em, especially if I just think ‘em’ I would be involved in it, so that is what I think” (IP7)

“Whether I would still be able to continue doing it after the first or second time or whether my attitude would not change, I don't yet know, I have definitely never not done that, naturally, I mean supporting dying in the context of palliative care, but, em, other parts (2) of my canon of values, I think I would do it (3) result unknown” (IP15)

### Context: Assessing the patient's situation

A central aspect for nursing professionals is the context “assessing the patient's situation.” It is essential for nurses to analyze the patient's overall situation, which may foster the wish to die. And thus, also to be able to assess for themselves the role as a nurse that they would assume in the process of assisted suicide. Continuity in the nursing care of patients enables nurses to establish a professional closeness to the patient that provides space for trusting conversations.

“So, em, the nurses are always the ones who spend most of their time in the patient's room, that is, em, (2) em, we are there around the clock, we are also there on days when other professional groups are not there, i.e., at the weekend or, em, at night or on public holidays and we are, em (2) we are also there in other situations, nursing is an occupation that involves, em, close physical contact, em, and for that reason I think that the relationship between a patient and the attending nurse is different to that between the patient and the attending doctor […], that other issues then also emerge […] so that, that definitely changes something and probably creates a certain familiarity” (IP7)

In this context, the interviewees also speak of a “good basis of trust” (IP4), which, in the view of the nursing professionals, is apparently necessary for patients to open up. The background knowledge generated by nursing professionals through conversations with patients serves to assess the extent to which they themselves can understand the wish to die and, related to this, whether and how intensively they want to support the process of assisted suicide.

“That's why I can't, I think that is something where one always, I (3) really can't, I can't say in general, I think I have to, em, decide from patient to patient, from situation to situation” (IP10)

It must be ensured that it is possible to decide on a case-by-case basis whether the respective nursing professional considers assisted suicide to be comprehensible for the patient in the respective situation, to be able to orient the nursing support and the corresponding extent accordingly. It seems that the nurse must be able to grasp the overall situation for him/herself to feel capable of making a decision.

“Em, and I would have to be involved, during a longer process, so I wouldn't just go somewhere today and say yes, this afternoon at 4 p.m., em, well, I would like to get to know the person, I would like to know what went on before with the patient, how the consultations were, and so on” (IP19)

At the same time, the interviewees are aware of the professional group's proximity to the patients, which enables them to take on responsible tasks in assisted suicide. According to the interviewees, these responsibilities are not mere delegation activities; rather, they must proffer an opinion in the decision-making process, based on the patient's situation.

“I don't just see myself as the person who puts on the syringe driver, and, em, presses on and off ((short laugh)), a lot of things happen before that, indeed a lot happens in the whole process” (IP9)

### Intervening condition: Expanding knowledge about assisted suicide

Specialist knowledge about assisted suicide and the processes involved is relevant for the interviewees, to be able to act adequately and competently.

“When I provide support for something like this, I would wish to be involved = i.e., that I am not ordered to sit down here now and watch, instead I want to know what is happening there, how it is happening, what medication is being taken, what it is that I can do” (IP13)

The nurses interviewed have specific ideas about the topics they require knowledge about. These include the preparation and follow-up of assisted suicide as well as the tasks carried out during the assisted suicide itself.

“And that it isn't just done helter-, when it is done, I mean if we now just, if we were to say let's do it, that it is not said in such a helter-skelter way, right, let's begin tomorrow, but that it would really be explained, first to the staff, how it is to be done, perhaps one could simply do a role play, how would the procedure go and what do I need to watch out for, whatever, how how how do I behave or not, em, and that I would approach the whole thing really professionally and not say, like it seemed just now in this case with the older gentleman with the stroke, that you go there and then something happens, and I don't know what it is and is it normal, no idea (2), but rather professional preparation” (IP11)

In addition, it becomes apparent that this knowledge is also considered necessary to be able to answer questions and remove uncertainties in interactions with the patient and their relatives.

“If I were, em, well prepared then, em, (2) I think then it would be easier, because perhaps the relatives have also been told, maybe they have also been told that there might be side effects and it might go this way or that way, when all of that is openly discussed then I have, I would have a completely different, em, approach, then I could reach the woman on her level and tell her that right now exactly that which was explained to her is going to happen and now this will happen and come let's see, and we'll do it together and so on, and that I would then be much better able to create a situation in which the relatives also feel in good hands and of course the dying person too, em, (2) yes, I believe it has a lot to do with knowing or not knowing” (IP19)

Another major topic is to know the effects of the lethal medication as well as the associated complications, and to be able to react professionally as a nurse.

“So that I don't just em, offer, em, the after-effects, for example the medication, as a possibility of assisted suicide, but that, for example, I also recognise when the medication is not working, […] and that people in the process of dying might suffer, that I recognise, and that I also know what is happening there, so that even when I work, for example, with medication and the patient is not well sedated in such a dying process, that it, even if it is successful, that the dying process as such is a torture, I must be able to recognise that, I must be aware of it, that is knowledge that I need to have” (IP18)

To be able to generate the knowledge described, the interviewees would like to have special training and learning opportunities.

“Here, too, we need training for nursing, we need training for family doctors ‘who’ say okay, in the case of this we need that, but once again the question at the outset is what should you do when this or that situation arises (3) education and information that takes pl- or should take place both on the nursing side and, em, the doctor's side” (IP16)

### Strategy: Tolerating (being able to tolerate) the desire to die

One of the strategies for action is to be able to tolerate the desire to die, regardless of one's own attitude toward assisted suicide. Initially, the interviewees acknowledge the desire to die, see that it exists in the patient, and seek to discuss the matter.

“I am certainly in a position, when I am charged with doing so, to communicate with people about it and, em (3) and also to leave it with them, so, rather like I also supported this woman, to ask well, what do you hope for, what do you want, and what do you wish for and so on (simply) so that's what's behind it” (IP19)

The “offer of a dialogue” (IP16) is seen as the first step in dealing with the desire to die. In doing so, the interviewees try to put their own views aside and place the patient's self-determination in the foreground. The desire to die that exists is thus tolerated from a professional perspective, although differentiation can be made if the path of assisted suicide does not correspond to the personal opinion regarding the situation at hand, and thus assisted suicide cannot be supported by certain nursing professionals.

“Well then, you have to put yourself in the em, patient's situation, I think, and if- if they think it is right or, em, want it in the institution and seek death, then I think I have to accept it” (IP3)

“And I also have to say- like with this gentleman now in the last case study, I can, em, I can accept that he wants to: or in the case of the mentally ill woman but em, I accept his decision but I can't myself em, go along with it yet in that moment” (IP10)

### Consequence: Being there for the person and supporting them as they are

As a consequence of the main phenomenon “being able to understand the desire to die,” the interviewees show that regardless of their personal attitude toward assisted suicide, there is an interest in supporting patients in assisted suicide. The initial idea for this is the nursing value of being there, that is, not leaving people alone with their wishes and needs as far as possible.

“For me it would of course have been my job to support him, because when someone is in my care home and I am a member of staff here then I care for the people until their death, under which circumstances they then die is first of all completely irrelevant […] because if he falls in the bathtub and hits his head, I can't come along and say there's too much blood here for me, I'm not going to do it, y'know, now you'll just have to die alone, y'know, em, I can't do that either, y'know, and em, em, from that point of view, em = em, if I'm already working in this institution, then I think it is very much my job to support him, even if I don't agree with it I'm constantly doing things that I don't agree with but they are simply part of my work” (IP8)

The extent to which the interviewee is involved in supporting the patient depends on the phenomenon of the comprehensibility of the desire to die. Consequently, support by nursing professionals is case-specific and varies in intensity accordingly. In connection with this, the interviewees see a range of actions that can be integrated in the nursing support of assisted suicide.

“Being there, being approachable, accompanying, em, the whole process, alleviating symptoms (2) hmm, the aftercare, em, preparing the corpse” (IP1)

In the context of being there and providing support, two further factors play a role. On the one hand, the nursing professionals interviewed focus on the self-determination of the patient, in that the nursing actions are oriented toward the wishes and ideas of the patient. The reason for this seems to be the idea that in the final moments of life, the focus should be on what the patient wants.

“Mm, so I would actually do it as I always do, see what the patient wants, how should it, how should things go, what can I do, in other words everything within the scope of my, my possibilities to make the whole thing as nice as possible, or, so for me the focus is always on these things what does the patient want (4), so always this, what would the patient like, how does he imagine it should be (2) and simply to see that I do my best or (2) do so much that it is good for the patient” (IP13)

On the other hand, in supporting patients before, during, and after the implementation of assisted suicide, they see their task in professional action to perceive changes in the patient's behavior and to react to them, regardless of their own attitude to assisted suicide. In these moments, the focus is on alleviating the patient's suffering, for example when complications arise after taking the lethal medicine.

“And then a situation arose that was something like an emergency, em, situation and in my opinion our role then is first to see that no suffering arises from it and I would simply, there was talk of restlessness and, em, nausea, vomiting, somehow, em, that's how I understood what was described, and I would try to, em, ease the suffering, to relieve him of that” (IP7)

## Discussion

The aim of this qualitative study was to describe the views and attitudes of nursing professionals from different care settings in the context of assisted suicide. It was possible to gain insights into which criteria the nursing professionals use in their assessment of assisted suicide. In addition, it was possible to find out which aspects guide the nursing professionals when it comes to the question of involving themselves as nursing professionals in assisting patients to commit suicide.

An international comparison shows that there are overlaps with study results concerned with the experience of desire for suicide assistance, as well as possible areas of nursing action. Denier et al. also show that Belgian nurses want to understand the wish to die before they become active in assisting suicide.^13^ Further, the nurses interviewed address similar areas of responsibility as well as fields of activity in assisted suicide that are also relevant to Dutch and Canadian nurses.

These include the discussion of the desire to die and related actions, dealing with the lethal medication, and caring for the bereaved.^13–17^ Among others, this overlaps with our findings in the categories “tolerating (being able to tolerate) the desire to die” (strategies), “being there for the person and supporting them as they are,” and “being aware of relatives' needs and supporting them where needed” (consequences). At the same time, there is one aspect that does not find agreement in comparable studies: The opinion formation of the nursing professionals interviewed for this study is not based on religious attitudes, but primarily on the phenomenon described.^18,19^

Based on these findings, there are central implications for the professional field of nursing in Germany. In essence, these implications amount to a recommendation to create a curricular offer for nursing professionals for the area of assisted suicide, to strengthen the attitude of nursing professionals toward assisted suicide, and to establish a legal standard for assisted suicide and medical professional groups such as nursing.

Professional groups that are increasingly approached in cases of requested death, such as the nursing profession, bear a great responsibility. They must, possibly as part of an interprofessional team, determine the cause of the desire to die and recognize whether there might be any ambivalence, that is, whether the wish to die is rather a wish for support in a life situation that is challenging for the patient, rather than an actual desire to die.

This requires experience as well as sensitivity in dealing with desires to die. The nursing profession can make a “specific contribution” (original quote in German) in this context.^20^ At the same time, nursing professionals who are confronted with desires to die initially feel insecure and inhibited in responding to such wishes.^21^ The Centre for Palliative Medicine at University Hospital Cologne has therefore developed, piloted, and evaluated a training concept for dealing with desires to die in the study “Desire to Die in Palliative Care—Optimization of Management” (DEDIPOM).^22^

The results of the DEDIPOM study show, among other things, that people who are confronted with desires to die experience increased confidence in the long term when dealing with desires to die.^22^ Thus, there are already empirical findings that prove the effectiveness of information, guidelines, and training with regard to desires to die. The study data analyzed here underline the existing evidence that nursing professionals gain confidence from such training.

However, desires to die are not identical to suicidality and thus to assisted suicide.^23^ Given the findings of the SEILASS study, information and training on how to deal with desires to die are not sufficient should nurses choose to provide support for assisted suicide. The nursing professionals explicitly demand to be informed about essential knowledge on the procedure of assisted suicide, the effectiveness of the lethal medication and possible complications.

However, various professional societies and experts in Germany focus on active suicide prevention, which is promoted by the state.^24,25^ In the context of the original work, it would appear sufficient not only to focus on suicide prevention, but also to ensure that nursing professionals involved in assisted suicide are given the confidence and competence to deal empathically and individually with patients and their relatives before, during, and after an assisted suicide.

The development of a viewpoint can have a supportive effect here. Kremeike et al. state that in the encounter with people who express a wish to die, it is essential to show an “open, interested and respectful attitude to the subjective lived reality” (original quote in German) of this person.^21^

In doing so, the motives and background for the desire to die should be taken on board without personal value judgments.^21^ To enable this, however, it is necessary to have the requisite basic attitude in the first place. Yet the nursing professionals in the SEILASS study demonstrate a vacillating attitude toward assisted suicide, which, in turn, influences the extent to which the desire to die can be understood.

It, therefore, seems to be essential to support nursing professionals in developing their own, solid viewpoint. In the long term, this supports nursing professionals in being able to react empathetically and as non-judgmentally as possible to the desire to die.

This is also necessary to ensure that patients who express a wish to die and request assisted suicide are not at the mercy of nurses who are critical of assisted suicide and reject the desire to die per se. At the same time, the development of a stance can be conducive to being able to deal with the emotional and ethically conflicted topic of assisted suicide and to find a basis for justifying one's own actions.

The demand, analyzed here, by nursing professionals for further education and training opportunities, specifically developed for dealing with assisted suicide, also appears essential because it is known from international studies that nursing professionals experience emotional consequences from long-term involvement in assisted suicide.^5,17^

Therefore, a thematic discussion of assisted suicide as well as adequate preparation for the activities involved in assisted suicide, especially with regard to situations in which complications arise, should be aimed for in advance.

Finally, the demand for legal regulation should also be emphasized at this point. Denier et al. describe the importance of the Belgian Euthanasia Act (2002) as a crucial factor for Belgian nurses to achieve security and positive feelings in the context of assisted suicide tasks.^13^ This is in line with statements made by nurses in the SEILASS study.

Clear and transparent guidelines that set out a framework of action for nursing professionals can help to give them a perspective on the possible fields of action and promote their self-confidence within these fields of action. Thus, the practical implications described are addressed not only to institutions and people who have direct contact with nursing issues and professional practice, but also to legislators.

### Limitations

With regard to the sample, it must be taken into account that only one nursing professional, particularly from the setting of long-term care, was included. Since suicide rates are increasing significantly among the elderly and very old,^26^ it would appear reasonable to strive for further research on assisted suicide with nursing professionals involved in long-term care.

It should also be noted that the nursing professionals interviewed have not yet been involved in assisted suicide themselves. In terms of the theory generated from the data, this means that it may change as assisted suicide becomes more established, and as nurses become more involved. In addition to supporting the results of this qualitative study, it would seem reasonable to add a quantitative approach.

## Conclusions

The design of a curricular offer with a focus on knowledge transfer as well as the promotion and consolidation of an attitude toward assisted suicide should be prioritized in Germany. Since nurses play a fundamental role in assisted suicide, the role of nurses should be considered, especially at the political level, and clear framework conditions should be created.

## Abbreviations Used

BVerfG: Bundesverfassungsgericht
DEDIPOM: Desire to Die in Palliative Care—Optimization of Management
SEILASS: Sichtweisen und Einstellungen von Pflegefachkräften unterschiedlicher Versorgungssettings zur Suizidassistenz
